# ﻿Pollen morphology and species differentiation in selected species of Inuleae (Asteraceae)

**DOI:** 10.3897/phytokeys.263.165364

**Published:** 2025-10-08

**Authors:** Tianmeng Qu, Xinyu Chen, Xinyi Zheng, Yanru Zhang, Yizhen Shao, Hongze Sun, Bing Zhang, Gan Xie, Zhixi Fu

**Affiliations:** 1 Key Laboratory of Land Resources Evaluation and Monitoring in Southwest, Sichuan Normal University, Ministry of Education, Chengdu 610066, China; 2 College of Life Sciences, Sichuan Normal University, Chengdu 610101, China; 3 College of Life Science, Henan Agricultural University, Zhengzhou 450000, China; 4 Beijing Dayu Middle School, Beijing 102300, China; 5 Sichuan Leshan Ecological Environment Monitoring Center Station, Leshan 614099, China; 6 Big Data and AI Research Center of Biodiversity Conservation, Institute of Botany, Chinese Academy of Sciences, Beijing 100093, China; 7 Sustainable Development Research Center of Resources and Environment of Western Sichuan, Sichuan Normal University, Chengdu, China

**Keywords:** *
Blumea
*, *
Carpesium
*, HCA, palynology, PCA, SEM, taxonomy

## Abstract

The tribe Inuleae is widely distributed within Asteraceae and exhibits considerable morphological variation, which complicates species classification. Pollen morphology provides relatively stable features for species delimitation, yet comprehensive palynological data for many species remain limited. In this study, the pollen morphology of 19 species from eight genera of Inuleae was investigated using light microscopy (LM) and scanning electron microscopy (SEM), with a focus on 10 quantitative traits. Multivariate analyses, including principal component analysis (PCA) and hierarchical clustering analysis (HCA), showed that both pollen size and exine ornamentation contribute to interspecific differentiation. Differences were especially notable between *Blumea* and *Carpesium*, with the latter showing larger pollen grains and more slender, scattered spines. This research also presents first-time palynological descriptions of *Blumea*, *Carpesium*, *Inula*, *Laggera*, *Pentanema*, and *Pterocaulon*. Overall, the findings indicate that pollen morphological traits are informative for species differentiation and lay a foundation for further palynological classification within Inuleae.

## ﻿Introduction

Inuleae (Asteraceae: Asteroideae), sensu Anderberg and Eldenäs (2007), comprises approximately 690 species in 66 genera and represents a major lineage of the family. Most species are subshrubs, shrubs, or perennial herbs, mainly adapted to warm or temperate climates, and the tribe is primarily distributed in Eurasia, with additional occurrences in North Africa, Asia, and adjacent Arabia (Anderberg 1989, 1991, 2009; Anderberg and Eldenäs 2007; Pornpongrungrueng et al. 2007). The tribe exhibits considerable morphological heterogeneity, particularly in style types, bract morphology, and pollen wall structure (Merxmüller et al. 1977; Nylinder and Anderberg 2015). Although Merxmüller et al. (1977) provided a systematic overview based on palynology, cytology, and morphology, Inuleae does not form a distinct monophyletic group, highlighting the complexity of its classification (Bremer 1987; Anderberg 1989; Kim and Jansen 1995).

Pollen morphology has long contributed to plant taxonomy, supporting species and genus identification across diverse plant groups (Erdtman 1952; Larson and Skvarla 1962; Nowicke and Skvarla 1979; Ferguson 1985). In large and taxonomically complex families such as Asteraceae, it also provides insights into intergeneric relationships and evolutionary trends (Skvarla et al. 1977, 2005; Zhao et al. 2000; Pereira Coutinho and Dinis 2007, 2009; Wortley et al. 2007; Osman 2011). Pollen morphology is generally genetically conserved and shows limited variation across environmental conditions, which enhances its reliability as a taxonomic character (Zarrei and Zarre 2005; Blackmore 2007; Kadluczka et al. 2022). Key palynological traits, including size, shape, aperture type, exine ornamentation, and perforation dimensions, are widely used to infer genetic relationships and support taxonomic classification (Heidarian et al. 2021; Li et al. 2021). Although pollen morphology tends to be conserved within genera of Asteraceae, subtle trait differences can still assist in distinguishing closely related species (El-Ghazaly and Anderberg 1995; Magenta et al. 2010; Carrijo et al. 2013; Huang et al. 2017; Coutinho et al. 2020; Marques et al. 2021).

Recent studies on the palynology of the tribe Inuleae have made notable progress. Inuleae pollen typically exhibits a caveate, echinate–microperforate exine (Leins 1971; Anderberg 2009), though subtle interspecific variations hold phylogenetic significance. For example, *Cyathocline* has smaller grains with longer spines compared to *Blumea* (Peng et al. 2023). The three morphotypes of *Pulicaria* were identified based on spine and sculpturing features (Coutinho et al. 2011). Previous work has emphasized the importance of exine morphology, particularly spine shape and sculpturing patterns, in species classification and in providing consistent morphological evidence for phylogenetic analyses across several genera (Osman 2006; Pereira Coutinho and Dinis 2007; Zarin et al. 2010; Karlıoğlu Kılıç et al. 2021). However, despite these advances, palynological research on Inuleae remains relatively sparse (Perveen 1999; Pornpongrungrueng et al. 2007; Bahadur et al. 2022), and the pollen characteristics of many species are still unclear.

Accordingly, this study focuses on 19 species of Inuleae to analyze pollen morphology and its systematic implications. Detailed pollen morphological data for 11 species are presented here for the first time. Species sampling targeted lineages within Inuleae considered informative for interpreting pollen characters, enabling lineage-aware interpretation among the sampled taxa. This study forms the second part of an ongoing investigation into palynological and evolutionary patterns in Asteraceae, following our work on Astereae (Qu et al. 2025). The objectives are to (i) provide palynological data for Inuleae species using light microscopy (LM) and scanning electron microscopy (SEM); (ii) distinguish species with similar morphology based on pollen traits; and (iii) evaluate the role of palynology in the taxonomy of this group.

## ﻿Materials and methods

### ﻿Sampling

Nineteen pollen samples were selected from voucher specimens deposited in the PE Herbarium at the Institute of Botany, Chinese Academy of Sciences. Sampling followed a tribe-level phylogenetic framework to cover major lineages of Inuleae. The selected taxa include species from *Blumea*, *Carpesium*, and all studied genera of the tribe (Table 1). Scientific names were standardized according to Plants of the World Online (https://powo.science.kew.org/, accessed 7 July 2025). We reviewed prior palynological literature to verify whether each species had been examined previously, and this information is summarized in Table 1.

**Table 1.** List of the voucher specimens in the PE Herbarium, Institute of Botany, Chinese Academy of Sciences.

Species	Subtribe (Fu et al. 2016)	Locality	Collection Date	Collector	Specimen barcodes	Palynological record
*Blumea balsamifera* (L.) DC.	Inulinae	Guizhou, China	10 Apr. 1959	Qiannan Team	PE 00569129	Peng et al. 2023
*Blumea megacephala* (Randeria) C.T.Chang & C.H.Yu	Inulinae	Guangxi, China	10 Dec. 2015	Z. Y. Zhang et al.	PE 02112569	Peng et al. 2023
*Blumea lacera* (Burm.f.) DC.	Inulinae	Guangxi, China	15 Apr. 1998	H. N. Qin et al.	PE 01997400	Peng et al. 2023
*Blumea fistulosa* (Roxb.) Kurz	Inulinae	Guangdong, China	5 Apr. 1997	Shenzhen Expedition Team	PE 01401511	Peng et al. 2023
*Blumea lanceolaria* (Roxb.) Druce	Inulinae	Guangxi, China	7 Nov. 2010	Y. S. Chen	PE 02110949	First report
*Blumea formosana* Kitam.	Inulinae	Jiangxi, China	7 Oct. 1980	Southern Grassland Team	PE 01776724	First report
*Carpesium szechuanense* F.H.Chen & C.M.Hu	Inulinae	Sichuan, China	8 Aug. 2007	Y. S. Chen	PE 01670507	First report
*Carpesium triste* Maxim.	Inulinae	Tochigi, Japan	28 Aug. 1988	M. Furuse	PE 01292635	First report
*Carpesium cordatum* F.H.Chen & C.M.Hu	Inulinae	Sichuan, China	13 Jul. 2005	D.E.Boufford et al.	PE 01882433	First report
*Carpesium cernuum* L.	Inulinae	Henan, China	Aug. 2009	Yuntai Mountain collection Team	PE 02015903	Zarin et al. 2010
*Carpesium longifolium* F.H.Chen & C.M.Hu	Inulinae	Sichuan, China	5 Sep. 2010	Gulin Expedition Team	PE 01864636	First report
*Inula japonica* Thunb.	Inulinae	Shaanxi, China	18 Jul. 1953	K. J. Fu	PE 00571428	First report
*Karelinia caspia* (Pall.) Less.	Plucheinae	Gansu, China	12 Aug. 1964	-	PE 01577784	Lu et al. 2018
*Laggera crispata* (Vahl) Hepper & J.R.I.Wood	Plucheinae	Guizhou, China	2 May 2003	G. F. Wang	PE 01688665	First report
*Laggera alata* (D. Don) Sch.Bip. ex Oliv.	Plucheinae	Guizhou, China	7 Apr. 2004	F. C. Wang	PE 01717274	Meo and Khan 2009
Pentanema indicum var. hypoleucum (Hand.-Mazz.) Y.Ling	Inulinae	Guizhou, China	15 Mar. 1960	Guizhou Team	PE 01711140	First report
*Pentanema cernuum* (Dalzell) Y.Ling	Inulinae	Yunnan, China	-	A. Henry	PE 01711084	First report
*Pterocaulon redolens* (Willd.) Fern.-Vill.	Plucheinae	Queensland, Australia	1 Dec. 2012	K. R. McDonald	PE 02110665	First report
*Pulicaria dysenterica* (L.) Bernh.	Inulinae	Istria, Slovenia	7 Sep. 2009	V. Mikolas et al.	PE 02012570	Coutinho et al. 2011

### ﻿Pollen preparation

Pollen samples were acetolysed using standard methods (Erdtman 1960) and fixed in glycerine jelly. Processing and observation under LM and SEM followed standard procedures (Wang et al. 1995). Pollen grains were observed and photographed at a magnification of ×600 under LM (Leica DM 4000) and at an acceleration voltage of 30 kV under SEM (Hitachi S-4800). Prior to SEM analysis, samples were sputter-coated with platinum to enhance image quality and ensure conductivity. Descriptions of pollen morphological traits follow the terminology systems of Hesse and Blackmore (2013) and Halbritter et al. (2018).

### ﻿Data acquisition

**Figure 1.**
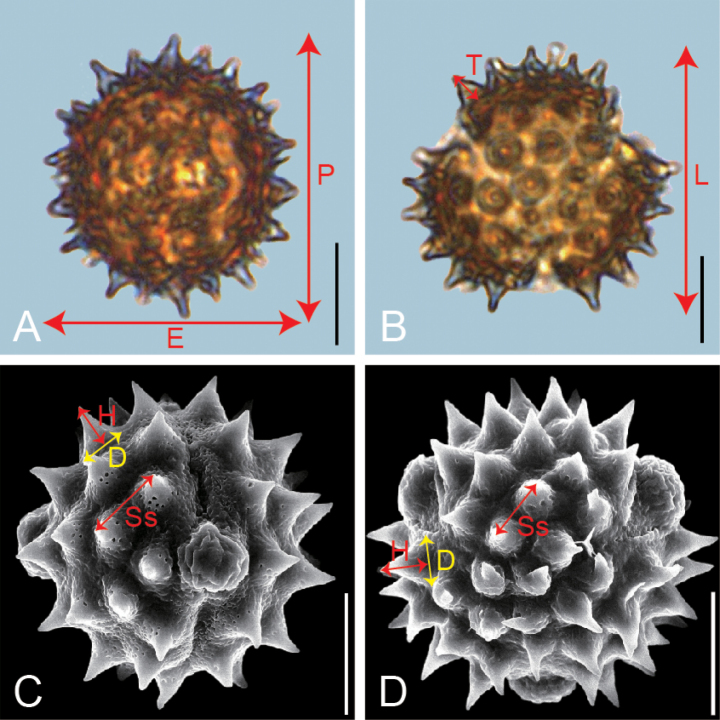
Graphical illustration of measured pollen morphological traits in Inuleae (A. *Inula japonica*; B. *Karelinia caspia*; C. *Blumea fistulosa*; D. *Carpesium cernuum*). The scale bar in the LM and SEM overview represents 10 µm.

As shown in Fig. 1, pollen morphological traits measured under LM included P: polar length in equatorial view; E: equatorial width in equatorial view; P/E; T: exine thickness in polar view; L: pollen length in polar view; and T/L. Each trait was measured for 20 pollen grains per species. The exine ornamentation traits measured under SEM included D: diameter of spine base; H: spine height; D/H; and Ss: spine spacing. For these four traits, measurements were taken on five pollen grains per trait, with four randomly selected regions per pollen grain, resulting in 20 measurements per trait (Wrońska-Pilarek et al. 2015; Lu et al. 2022). Pollen shape types were determined based on P/E ratios, following the definitions of Erdtman (1969) and Wang et al. (1995). Grains were categorized as perprolate (P/E > 2), prolate (1.32 < P/E ≤ 2), subprolate (1.14 < P/E ≤ 1.32), or spherical (0.88 < P/E ≤ 1.14). Descriptive morphological terminology follows Punt et al. (2007). Mean (M) and standard deviation (SD) values for 10 pollen traits were calculated across 19 species (Table 2, Suppl. material 1).

**Table 2.** Pollen morphological traits of 19 selected species (Ss: spine spacing; D: diameter of spine base; H: spine height; P: polar length in equatorial view; E: equatorial width in equatorial view; T: exine thickness in polar view; L: pollen length in polar view).

Species	Ss (μm)	D (μm)	H (μm)	D/H	P (μm)	E (μm)	P/E	T (μm)	L (μm)	T/L
*Blumea balsamifera* (L.) DC.	4.79±0.23	3.39±0.12	3.58±0.17	0.95±0.03	28.82±1.48	26.86±1.12	1.07±0.04	4.27±0.27	28.04±1.29	0.15±0.01
*Blumea megacephala* (Randeria) C.T.Chang & C.H.Yu	6.01±0.55	3.69±0.36	3.95±0.36	0.93±0.04	26.68±1.66	24.76±1.21	1.08±0.04	4.10±0.30	27.50±1.29	0.15±0.01
*Blumea lacera* (Burm.f.) DC.	4.80±0.43	3.00±0.17	3.98±0.23	0.75±0.04	25.70±1.48	25.54±0.97	1.01±0.05	4.09±0.26	27.07±1.57	0.15±0.01
*Blumea fistulosa* (Roxb.) Kurz	4.92±0.42	3.31±0.34	3.86±0.40	0.86±0.06	29.94±1.67	27.58±1.58	1.09±0.03	4.05±0.18	30.20±1.10	0.13±0.00
*Blumea lanceolaria* (Roxb.) Druce	5.41±0.39	3.13±0.27	3.61±0.24	0.87±0.04	27.72±0.67	25.73±0.64	1.08±0.02	3.96±0.23	28.23±1.60	0.14±0.01
*Blumea formosana* Kitam.	5.69±0.24	3.26±0.19	4.09±0.24	0.80±0.03	32.90±1.66	29.79±1.50	1.10±0.02	4.14±0.13	30.35±1.37	0.14±0.01
*Carpesium szechuanense* F.H.Chen & C.M.Hu	5.23±0.44	2.96±0.22	4.34±0.33	0.68±0.03	31.43±0.97	28.86±0.90	1.09±0.02	4.53±0.19	31.55±1.41	0.14±0.00
*Carpesium triste* Maxim.	5.08±0.27	2.96±0.31	3.74±0.38	0.79±0.04	31.60±0.83	29.12±0.70	1.09±0.02	4.06±0.21	30.16±1.55	0.13±0.00
*Carpesium cordatum* F.H.Chen & C.M.Hu	5.29±0.23	3.16±0.16	4.11±0.22	0.77±0.04	34.34±1.00	32.16±0.97	1.07±0.01	4.66±0.12	33.49±0.84	0.14±0.00
*Carpesium cernuum* L.	5.70±0.32	3.47±0.13	4.32±0.24	0.80±0.04	31.73±0.82	28.82±0.66	1.10±0.02	4.56±0.17	30.95±1.13	0.15±0.00
*Carpesium longifolium* F.H.Chen & C.M.Hu	6.34±0.51	3.75±0.35	5.17±0.55	0.73±0.04	35.06±1.41	32.42±1.70	1.08±0.03	5.12±0.18	32.98±1.20	0.16±0.01
*Inula japonica* Thunb.	5.06±0.33	2.90±0.24	3.51±0.27	0.83±0.03	27.51±0.74	26.23±0.91	1.05±0.02	3.86±0.13	27.06±1.04	0.14±0.00
*Karelinia caspia* (Pall.) Less.	5.62±0.23	3.28±0.13	3.79±0.17	0.87±0.03	31.28±0.81	27.69±0.90	1.13±0.02	4.33±0.21	29.65±1.19	0.15±0.00
*Laggera crispata* (Vahl) Hepper & J.R.I.Wood	5.76±0.17	3.27±0.19	3.83±0.14	0.85±0.03	27.19±1.20	25.51±1.15	1.07±0.02	3.94±0.21	27.97±0.82	0.14±0.01
*Laggera alata* (D. Don) Sch.Bip. ex Oliv.	4.65±0.26	2.75±0.16	3.22±0.13	0.85±0.03	27.19±1.42	24.51±1.33	1.11±0.03	3.75±0.22	26.62±0.93	0.14±0.01
Pentanema indicum var. hypoleucum (Hand.-Mazz.) Y.Ling	4.38±0.27	2.52±0.18	2.91±0.24	0.87±0.02	23.51±0.91	22.39±0.75	1.05±0.03	3.18±0.19	23.26±0.73	0.14±0.01
*Pentanema cernuum* (Dalzell) Y.Ling	4.51±0.18	2.60±0.10	2.96±0.14	0.88±0.03	22.64±0.92	21.36±0.82	1.06±0.03	3.20±0.14	23.05±1.06	0.14±0.01
*Pterocaulon redolens* (Willd.) Fern.-Vill.	4.42±0.21	2.42±0.10	2.69±0.11	0.90±0.02	22.77±1.56	22.00±1.52	1.04±0.02	3.06±0.12	22.67±1.31	0.14±0.01
*Pulicaria dysenterica* (L.) Bernh.	4.22±0.29	2.56±0.16	3.32±0.13	0.77±0.05	23.85±1.22	22.29±1.18	1.07±0.02	3.06±0.18	23.69±1.68	0.13±0.01

### ﻿Data analysis

Boxplots of the 10 pollen traits were generated using OriginPro 2025. To eliminate dimensional effects and improve comparability, trait data were standardized using Z-scores (Andrade 2021). Principal component analysis (PCA) was performed with the prcomp function in R (R Core Team. 2019) to reduce dimensionality, identify trait correlations, and determine the variables contributing most to total variance. PCA projections were visualized using ggplot2. One-way analysis of variance (ANOVA) and Pearson’s correlation analysis were carried out in SPSS v26.0 (IBM Corp., Armonk, NY) to test interspecific differences in trait means and generate a correlation matrix. Hierarchical cluster analysis (HCA) was conducted in OriginPro 2025 based on 10 quantitative palynological traits (Ss, D, H, D/H, P, E, P/E, T, L, and T/L), with Euclidean distances calculated and clustering performed using Ward’s method (Ye et al. 2015).

## ﻿Results

### ﻿Pollen morphological characteristics

**Figure 2.**
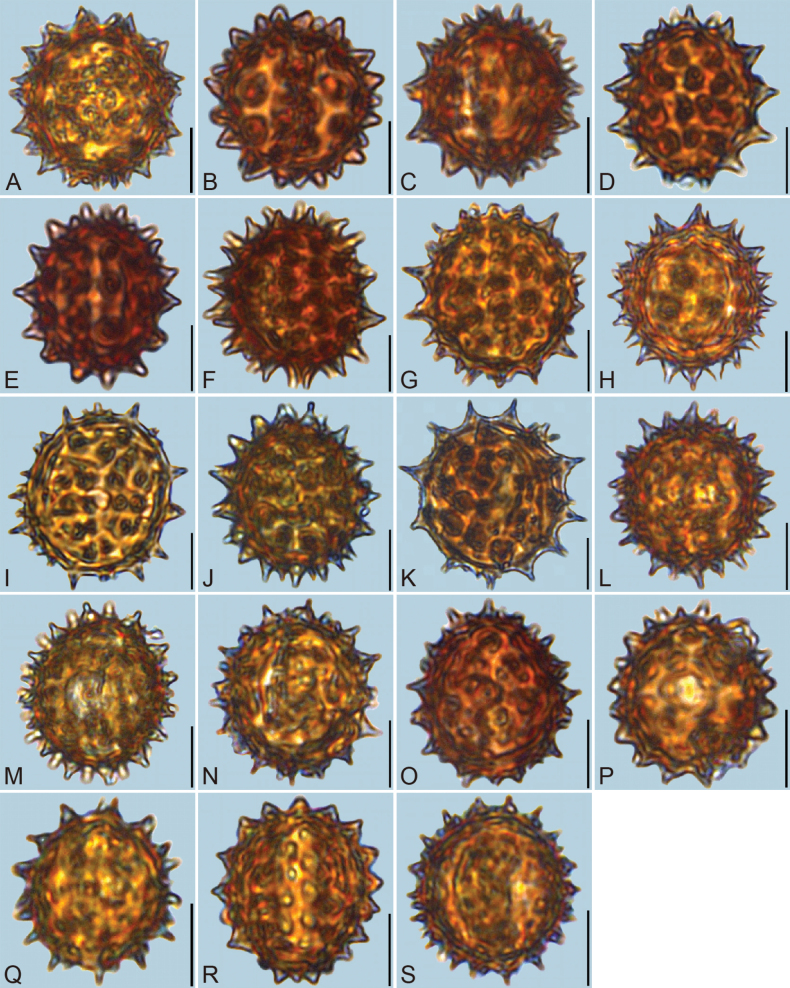
LM micrographs of pollen grains in equatorial view. A. *Blumea balsamifera*; B. *B. megacephala*; C. *B. lacera*; D. *B. fistulosa*; E. *B. lanceolaria*; F. *B. formosana*; G. *Carpesium szechuanense*; H. *C. triste*; I. *C. cordatum*; J. *C. cernuum*; K. *C. longifolium*; L. *Inula japonica*; M. *Karelinia caspia*; N. *Laggera crispata*; O. *L. alata*; P. Pentanema indicum var. hypoleucum; Q. *P. cernuum*; R. *Pterocaulon redolens*; S. *Pulicaria dysenterica*. Scale bars represents 10 µm.

**Figure 3.**
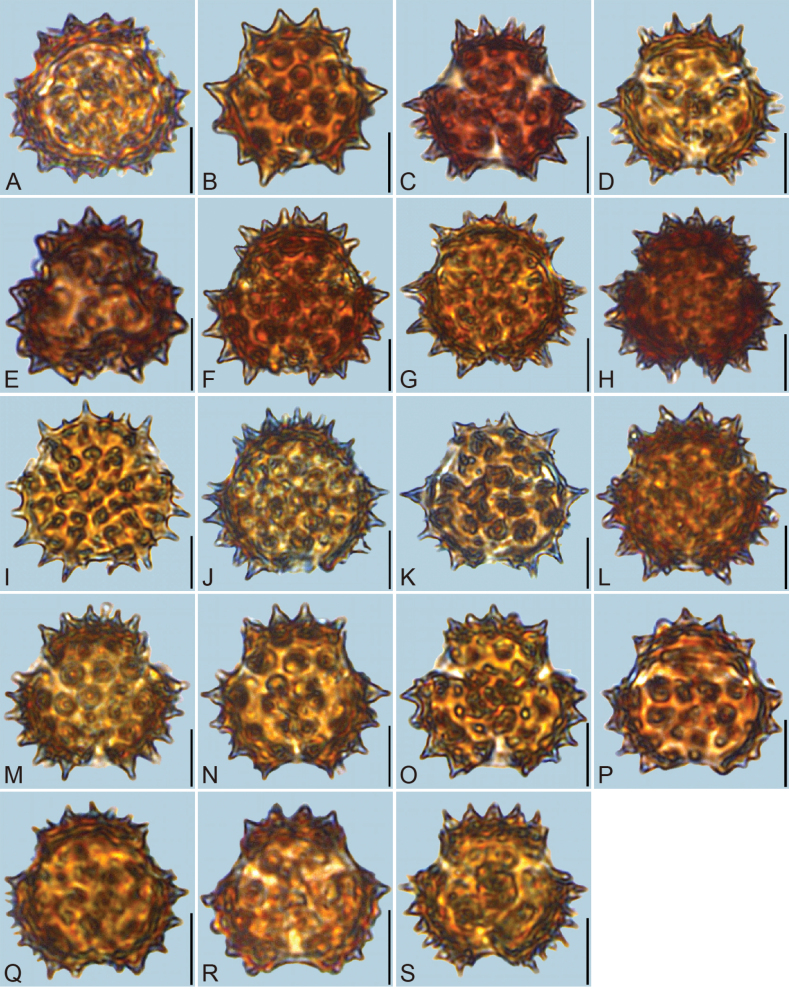
LM micrographs of pollen grains in polar view. A. *Blumea balsamifera*; B. *B. megacephala*; C. *B. lacera*; D. *B. fistulosa*; E. *B. lanceolaria*; F. *B. formosana*; G. *Carpesium szechuanense*; H. *C. triste*; I. *C. cordatum*; J. *C. cernuum*; K. *C. longifolium*; L. *Inula japonica*; M. *Karelinia caspia*; N. *Laggera crispata*; O. *L. alata*; P. Pentanema indicum var. hypoleucum; Q. *P. cernuum*; R. *Pterocaulon redolens*; S. *Pulicaria dysenterica*. The scale bar represents 10 µm.

**Figure 4.**
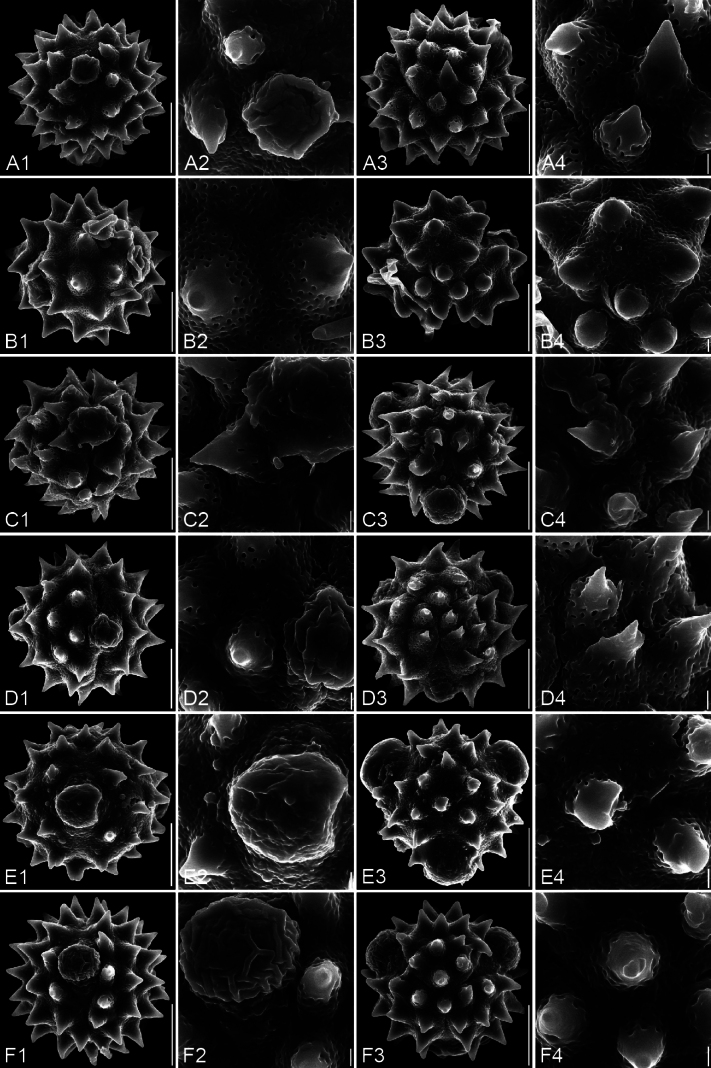
SEM micrographs of pollen grains. For each species, four images are arranged in a row: 1. Equatorial view; 2. Equatorial detail; 3. Polar view; 4. Polar detail. A1–A4. *Blumea balsamifera*; B1–B4. *B. megacephala*; C1–C4. *B. lacera*; D1–D4. *B. fistulosa*; E1–E4. *B. lanceolaria*; F1–F4. *B. formosana*. The scale bars represent 10 µm in overviews and 1 µm in details.

**Figure 5.**
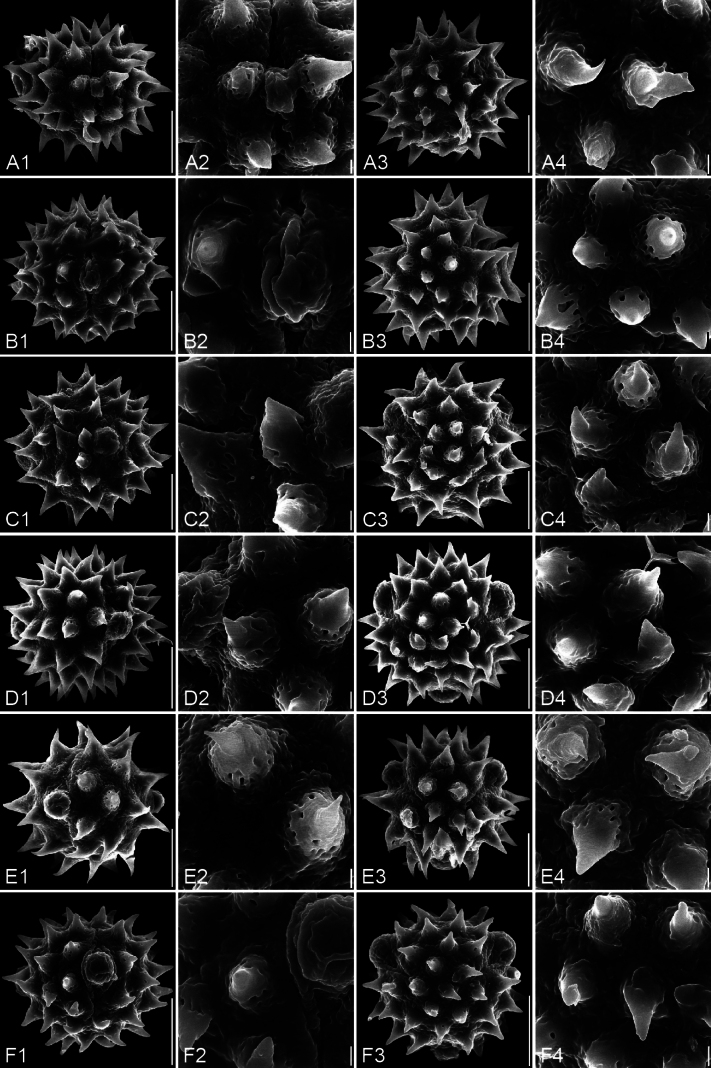
SEM micrographs of pollen grains. For each species, four images are arranged in a row: 1. Equatorial view; 2. Equatorial detail; 3. Polar view; 4. Polar detail. A1–A4. *Carpesium szechuanense*; B1–B4. *C. triste*; C1–C4. *C. cordatum*; D1–D4. *C. cernuum*; E1–E4. *C. longifolium*; F1–F4. *Inula japonica*. The scale bars represent 10 µm in overviews and 1 µm in details.

**Figure 6.**
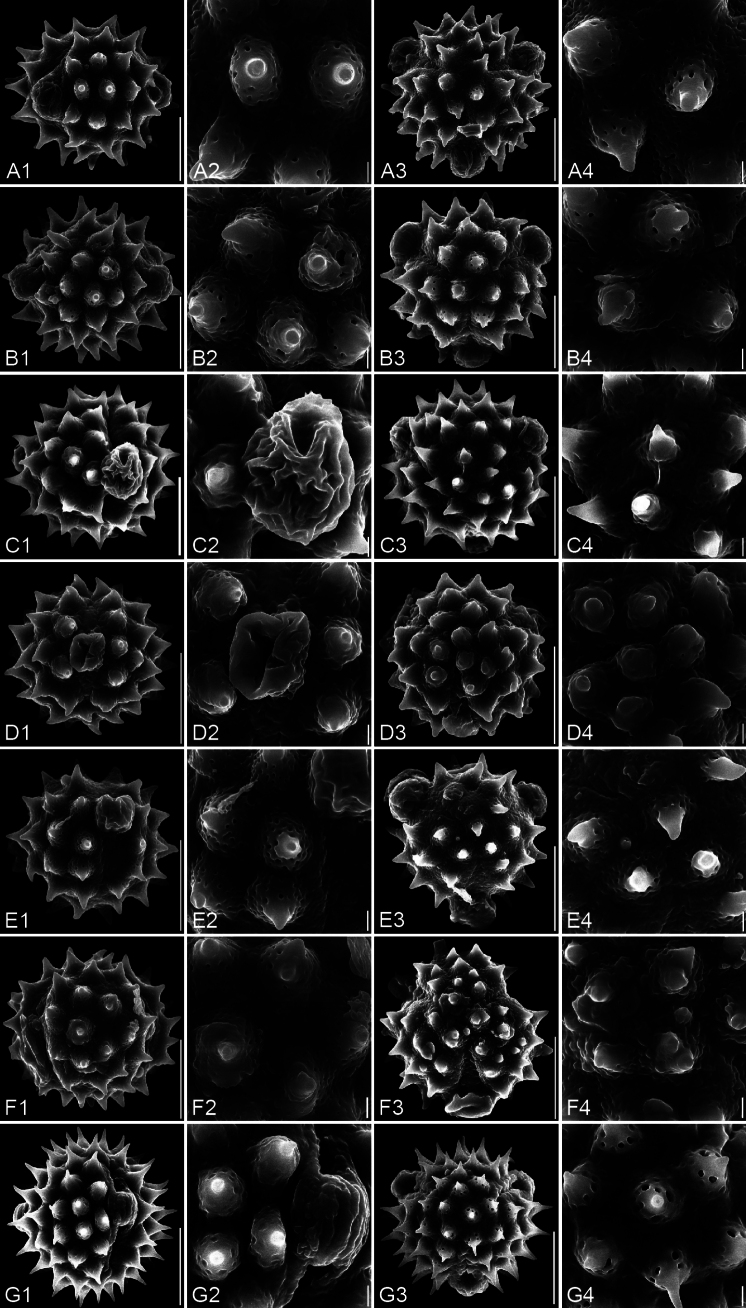
SEM micrographs of pollen grains. For each species, four images are arranged in a row: 1. Equatorial view; 2. Equatorial detail; 3. Polar view; 4. Polar detail. A1–A4. *Karelinia caspia*; B1–B4. *Laggera crispata*; C1–C4. *L. alata*; D1–D4. Pentanema indicum var. hypoleucum; E1–E4. *P. cernuum*; F1–F4. *Pterocaulon redolens*; G1–G4. *Pulicaria dysenterica*. The scale bars represent 10 µm in overviews and 1 µm in details.

**Figure 7.**
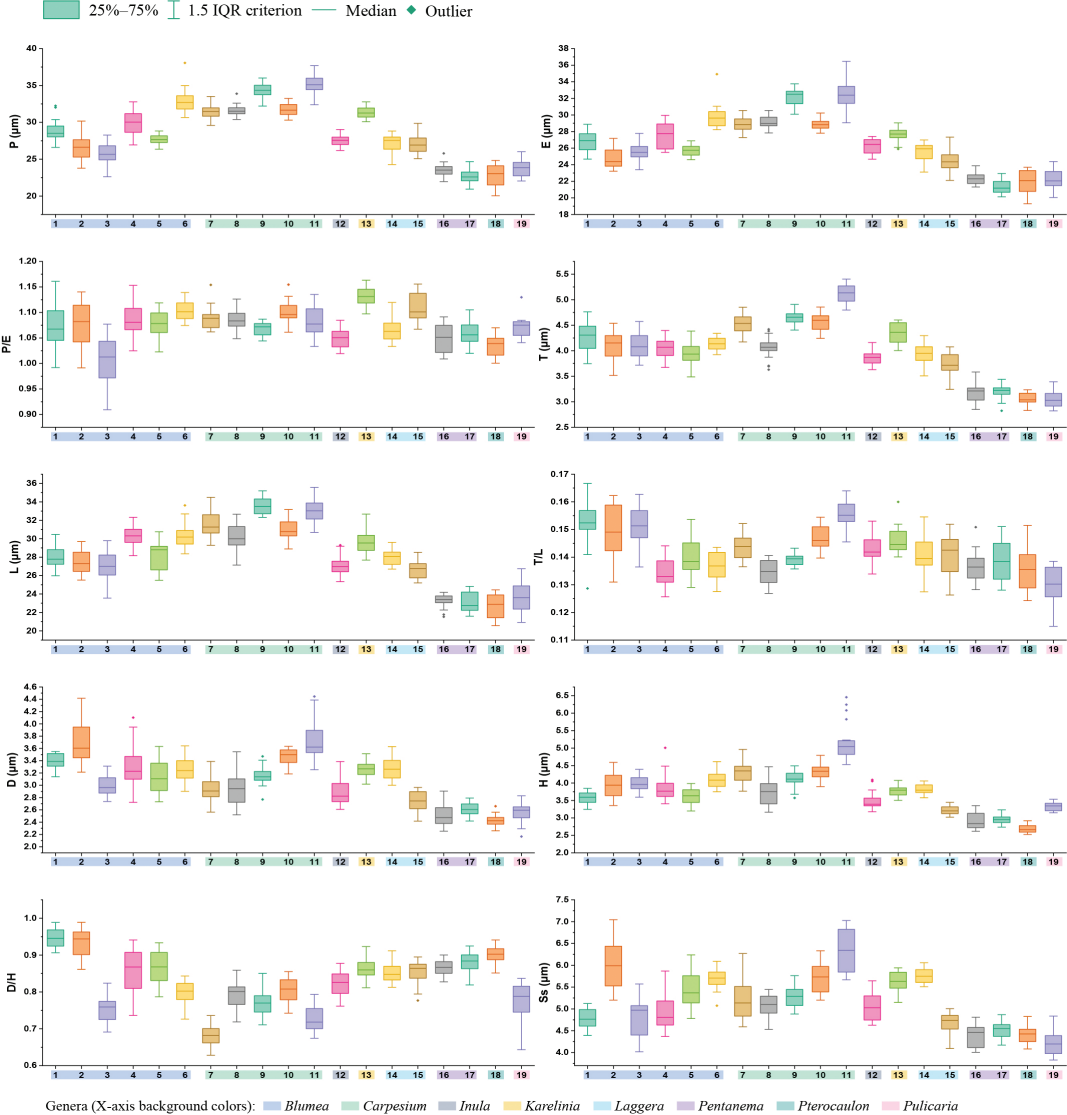
Boxplots of 19 sampled taxa showing the variations in pollen morphological traits (P. polar length in equatorial view; E. equatorial width in equatorial view; T. exine thickness in polar view; L. pollen length in polar view; D. diameter of spine base; H. spine height; Ss. spine spacing). 1. *Blumea balsamifera*. 2. *B. megacephala*. 3. *B. lacera*. 4. *B. fistulosa*. 5. *B. lanceolaria*. 6. *B. formosana*. 7. *Carpesium szechuanense*. 8. *C. triste*. 9. *C. cordatum*. 10. *C. cernuum*. 11. *C. longifolium*. 12. *Inula japonica*. 13. *Karelinia caspia*. 14. *Laggera crispata*. 15. *L. alata*. 16. Pentanema indicum var. hypoleucum. 17. *P. cernuum*. 18. *Pterocaulon redolens*. 19. *Pulicaria dysenterica*.

Detailed pollen morphological data observed under LM and SEM are presented in Figs 2–6. Quantitative values for 10 pollen traits across species are summarized in Table 2, with the mean ± standard deviation (M ± SD) reported for each trait. Boxplots in Fig. 7 illustrate the distribution patterns of these data, highlighting the interquartile range (25%–75%).

### ﻿Pollen shape and apertures

Pollen grains were generally spherical, with all species exhibiting tricolporate apertures visible under both LM and SEM. The P/E ratio (polar axis length / equatorial axis length) ranged from 1.01 to 1.13. In equatorial view, P ranged from 22.64 to 35.06 µm, and E from 21.36 to 32.42 µm. The T/L ratio (exine thickness / pollen length) ranged from 0.13 to 0.16. In polar view, T ranged from 3.06 to 5.12 µm, and L from 22.67 to 33.49 µm. Interspecific differences were observed in P, E, P/E, T, L, and T/L (p < 0.01).

Detailed measurements of pollen traits show variation across species within each genus.

#### ﻿*Blumea* DC.

In *Blumea*, the pollen grains have a polar length (P) ranging from 25.70 to 32.90 μm and an equatorial width (E) ranging from 24.76 to 29.79 μm, giving a P/E ratio between 1.01 and 1.10, the lowest observed in this study. The exine thickness (T) ranges from 3.96 to 4.27 μm, and the pollen length (L) ranges from 27.07 to 30.35 μm, with a T/L ratio between 0.13 and 0.15. These traits suggest that *Blumea* species have relatively large pollen grains compared to other genera.

##### *Carpesium* L.

In *Carpesium*, the pollen grains have a P ranging from 31.43 to 35.06 μm and an E from 28.82 to 32.42 μm, giving a P/E ratio between 1.07 and 1.10. The T ranges from 4.06 to 5.12 μm, and the L ranges from 30.16 to 33.49 μm, with a T/L ratio between 0.13 and 0.16. These traits suggest that *Carpesium* has relatively large pollen grains, with the largest P, E, T, and L values observed, surpassing most other genera measured in this study.

#### ﻿*Inula* L. s.str.

In *Inula*, the pollen grains have a P of 27.51 μm and an E of 26.23 μm, giving a P/E ratio of 1.05. The T is 3.86 μm, and the L is 27.06 μm, with a T/L ratio of 0.14. These traits suggest that *Inula* has moderately sized pollen grains in relation to the species measured.

#### ﻿*Karelinia* Less.

In *Karelinia*, the pollen grains have a P of 31.28 μm and an E of 27.69 μm, giving a P/E ratio of 1.13. The T is 4.33 μm, and the L is 29.65 μm, with a T/L ratio of 0.15. These traits suggest that *Karelinia* has relatively large pollen grains, with the largest P/E ratio observed in this study.

#### ﻿*Laggera* Sch.Bip. ex Benth. & Hook.f.

In *Laggera*, the pollen grains have a P of 27.19 μm and an E ranging from 24.51 to 25.51 μm, giving a P/E ratio between 1.07 and 1.11. The T ranges from 3.75 to 3.94 μm, and the L ranges from 26.62 to 27.97 μm, with a T/L ratio of 0.14. These traits suggest that *Laggera* has moderate-sized pollen grains, characteristic of the species measured.

#### ﻿*Pentanema* Cass.

In *Pentanema*, the pollen grains have a P ranging from 22.64 to 23.51 μm and an E from 21.36 to 22.39 μm, giving a P/E ratio between 1.05 and 1.06. The T ranges from 3.18 to 3.20 μm, and the L ranges from 23.05 to 23.26 μm, with a T/L ratio of 0.14. These traits suggest that *Pentanema* has relatively small pollen grains, with the smallest P and E values measured in this study.

#### ﻿*Pterocaulon* Elliott

In *Pterocaulon*, the pollen grains have a P of 22.77 μm and an E of 22.00 μm, giving a P/E ratio of 1.04. The T is 3.06 μm, and the L is 22.67 μm, with a T/L ratio of 0.14. These traits suggest that *Pterocaulon* has relatively small pollen grains, with the smallest T and L values measured in this study.

#### ﻿*Pulicaria* Gaertn.

In *Pulicaria*, the pollen grains have a P of 23.85 μm and an E of 22.29 μm, giving a P/E ratio of 1.07. The T is 3.06 μm, and the L is 23.69 μm, with a T/L ratio of 0.13. These traits suggest that *Pulicaria* has relatively small pollen grains relative to other genera measured.

### ﻿Pollen exine ornamentation

SEM observations revealed echinate exine surfaces, with spines tapering to sharp apices and typically surrounded by one or more rows of perforations at the base (Suppl. material 1). Inter-spinal regions were perforated. Spine base diameter (D) ranged from 2.42 to 3.75 µm, spine height (H) from 2.69 to 5.17 µm, and the D/H ratio from 0.68 to 0.95. Spine spacing (Ss) varied between 4.22 and 6.34 µm. Interspecies differences were observed in D, H, D/H, and Ss (p < 0.01).

Detailed measurements of pollen traits show variation across species within each genus.

#### ﻿*Blumea* DC.

In *Blumea*, the exine spines have a diameter (D) ranging from 3.00 to 3.69 μm and a height (H) between 3.58 and 4.09 μm. The D/H ratio ranges from 0.75 to 0.95, with the highest value of 0.95, indicating a relatively high height-to-diameter ratio compared to other genera. The spine spacing (Ss) ranges from 4.79 to 6.01 μm, showing a moderately wide distribution. These traits suggest that *Blumea* species have spines with a relatively high height-to-diameter ratio and moderate spacing.

#### ﻿*Carpesium* L.

In *Carpesium*, the exine spines have a D ranging from 2.96 to 3.75 μm and an H from 3.74 to 5.17 μm. The D/H ratio ranges from 0.68 to 0.80, with the lowest value observed among the 19 species, indicating relatively slender spines. The Ss ranges from 5.08 to 6.34 μm, with the highest value observed in this study. These traits suggest that *Carpesium* has more slender spines with a wider spacing.

#### ﻿*Inula* L. s.str.

In *Inula*, the exine spines have a D of 2.90 μm and an H of 3.51 μm. The D/H ratio is 0.83, indicating a moderate ratio between height and diameter. The Ss is 5.06 μm. These traits suggest that *Inula* has relatively small spines with moderate spacing.

#### ﻿*Karelinia* Less.

In *Karelinia*, the exine spines have a D of 3.28 μm and an H of 3.79 μm, resulting in a D/H ratio of 0.87. The Ss is 5.62 μm, one of the larger values observed. These traits suggest that *Karelinia* has moderately long spines with relatively wide spacing compared to other species.

#### ﻿*Laggera* Sch.Bip. ex Benth. & Hook.f.

In *Laggera*, the exine spines have a D ranging from 2.75 to 3.27 μm and an H from 3.22 to 3.83 μm, resulting in a D/H ratio of 0.85. The Ss ranges from 4.65 to 5.76 μm. These traits suggest that *Laggera* has moderately long spines with evenly spaced distribution.

#### ﻿*Pentanema* Cass.

In *Pentanema*, the exine spines have a D from 2.52 to 2.60 μm and an H from 2.91 to 2.96 μm, resulting in a D/H ratio from 0.87 to 0.88, indicating short spines. The Ss ranges from 4.38 to 4.51 μm, with relatively narrow spacing. These traits suggest that *Pentanema* has small, compact spines with dense distribution.

#### ﻿*Pterocaulon* Elliott

In *Pterocaulon*, the exine spines have a D of 2.42 μm and an H of 2.69 μm, resulting in a D/H ratio of 0.90. The Ss is 4.42 μm. The spines in this genus are relatively small and tightly spaced, which contributes to a compact and dense ornamentation pattern on the pollen surface.

#### ﻿*Pulicaria* Gaertn.

In *Pulicaria*, the exine spines have a D of 2.56 μm and an H of 3.32 μm, resulting in a D/H ratio of 0.77. The Ss is 4.22 μm, with tight spacing. These traits suggest that *Pulicaria* has moderately long spines with a dense distribution.

### ﻿Multivariate analyses

PCA revealed that the first two principal components accounted for 81.06% of total morphological variation (Table 3). PC1 explained 67.39% of the variation and was primarily associated with size-related traits (P, E, L, and T). PC2 accounted for 13.67% and was influenced mainly by exine ornamentation traits, particularly D/H and T/L. Pearson’s correlation analysis showed strong positive correlations among the size-related traits (r > 0.90, p < 0.01). Spine height (H) was positively correlated with spine base diameter (D) but negatively correlated with D/H. Spine spacing (Ss) was significantly positively correlated with most size-related traits. The complete correlation matrix is provided in Table 4.

**Table 3.** Palynological characters used in the multivariate analysis of the Inuleae (P: polar length in equatorial view; E: equatorial width in equatorial view; T: exine thickness in polar view; L: pollen length in polar view; D: diameter of spine base; H: spine height; Ss: spine spacing). The contribution of every character for Axis 1 and Axis 2 is indicated.

	Character	Axis 1	Axis 2
1	P (μm)	0.939	-0.232
2	E (μm)	0.936	-0.244
3	P/E	0.482	-0.049
4	T (μm)	0.979	0.053
5	L (μm)	0.957	-0.192
6	T/L	0.560	0.611
7	D (μm)	0.854	0.451
8	H (μm)	0.950	-0.050
9	D/H	-0.452	0.737
10	Ss (μm)	0.851	0.299

**Table 4.** Correlation matrix of palynological characters in the Inuleae (P: polar length in equatorial view; E: equatorial width in equatorial view; T: exine thickness in polar view; L: pollen length in polar view; D: diameter of spine base; H: spine height; Ss: spine spacing). p < 0.05 (*), p < 0.01 (**).

	P (μm)	E (μm)	P/E	T (μm)	L (μm)	T/L	D (μm)	H (μm)	D/H	Ss (μm)
P (μm)	1	0.985**	0.559*	0.906**	0.971**	0.273	0.693**	0.835**	-0.469*	0.702**
E (μm)	0.985**	1	0.408	0.919**	0.972**	0.294	0.683**	0.857**	-0.517*	0.683**
P/E	0.559*	0.408	1	0.386	0.485*	-0.008	0.394	0.304	0.003	0.432
T (μm)	0.906**	0.919**	0.386	1	0.943**	0.557*	0.832**	0.921**	-0.417	0.787**
L (μm)	0.971**	0.972**	0.485*	0.943**	1	0.272	0.740**	0.878**	-0.491*	0.725**
T/L	0.273	0.294	-0.008	0.557*	0.272	1	0.589**	0.498*	0.024	0.565*
D (μm)	0.693**	0.683**	0.394	0.832**	0.740**	0.589**	1	0.814**	-0.018	0.875**
H (μm)	0.835**	0.857**	0.304	0.921**	0.878**	0.498*	0.814**	1	-0.588**	0.811**
D/H	-0.469*	-0.517*	0.003	-0.417	-0.491*	0.024	-0.018	-0.588**	1	-0.176
Ss (μm)	0.702**	0.683**	0.432	0.787**	0.725**	0.565*	0.875**	0.811**	-0.176	1

**Figure 8.**
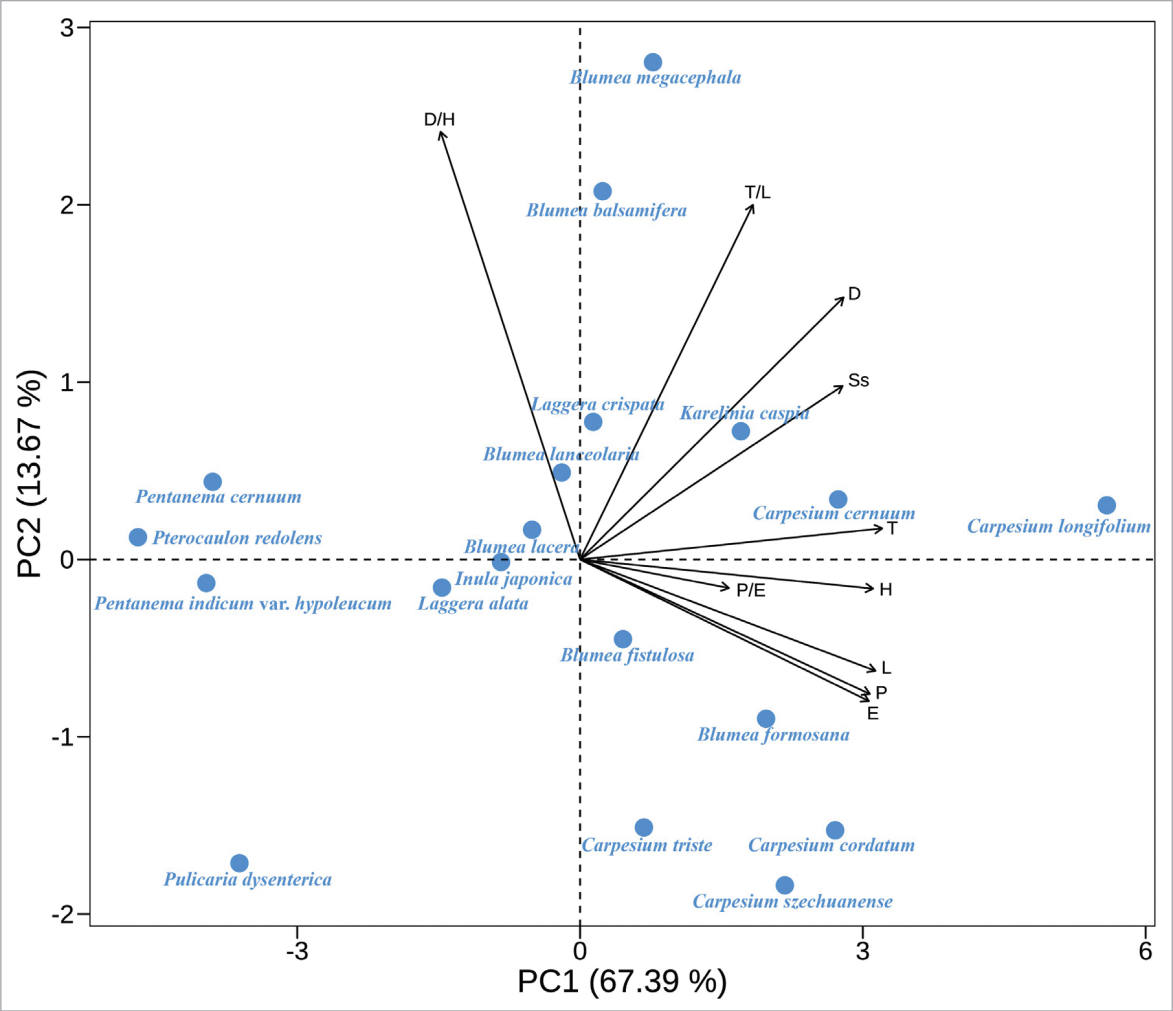
Principal Components Analysis graph showing the contribution of the ten attributes to explain variation in pollen grains of the studied Inuleae species; P. Polar length in equatorial view; E. Equatorial width in equatorial view, P/E, T. Exine thickness in polar view, L. Pollen length in polar view, T/L, D. Diameter of spine base, H. Spine height, D/H, Ss. Spine spacing.

As shown in Fig. 8, species along the positive PC1 axis (e.g., *Carpesium longifolium*, *C. cernuum*) exhibited larger pollen grains with narrow-conical spines, while species on the negative PC1 axis (e.g., *Pulicaria dysenterica*, *Pentanema cernuum*) showed smaller grains with less pointed spines. These morphological differences are supported by the detailed trait descriptions in the Suppl. material 1. PC2 separated species based on exine ornamentation, with species like *Blumea megacephala* and *B. balsamifera* exhibiting higher D/H and T/L values along the positive axis, while *C. szechuanense*, *C. triste*, and *C. cordatum* occupied negative PC2 values, reflecting contrasting exine characteristics. A central cluster, including *B. lacera* and *Inula japonica*, was positioned near the origin, suggesting moderate variability in both pollen size and exine ornamentation. The arrow lengths in Fig. 8 indicate sufficient variable loading for most traits, except for P/E. Notably, D/H showed a negative correlation with other traits, indicating that species with more elongated spines tended to have smaller pollen grains. The matrix of feature correlations (Table 4) supports these observations. *Pulicaria dysenterica*, positioned at the extreme negative ends of both PC1 and PC2, presented markedly small pollen grains and distinct ornamentation features, possibly representing an outlier relative to the main cluster. Overall, the PCA indicated that pollen size traits account for the majority of observed variation, while exine ornamentation provides complementary dimensions for interspecific differentiation.

**Figure 9.**
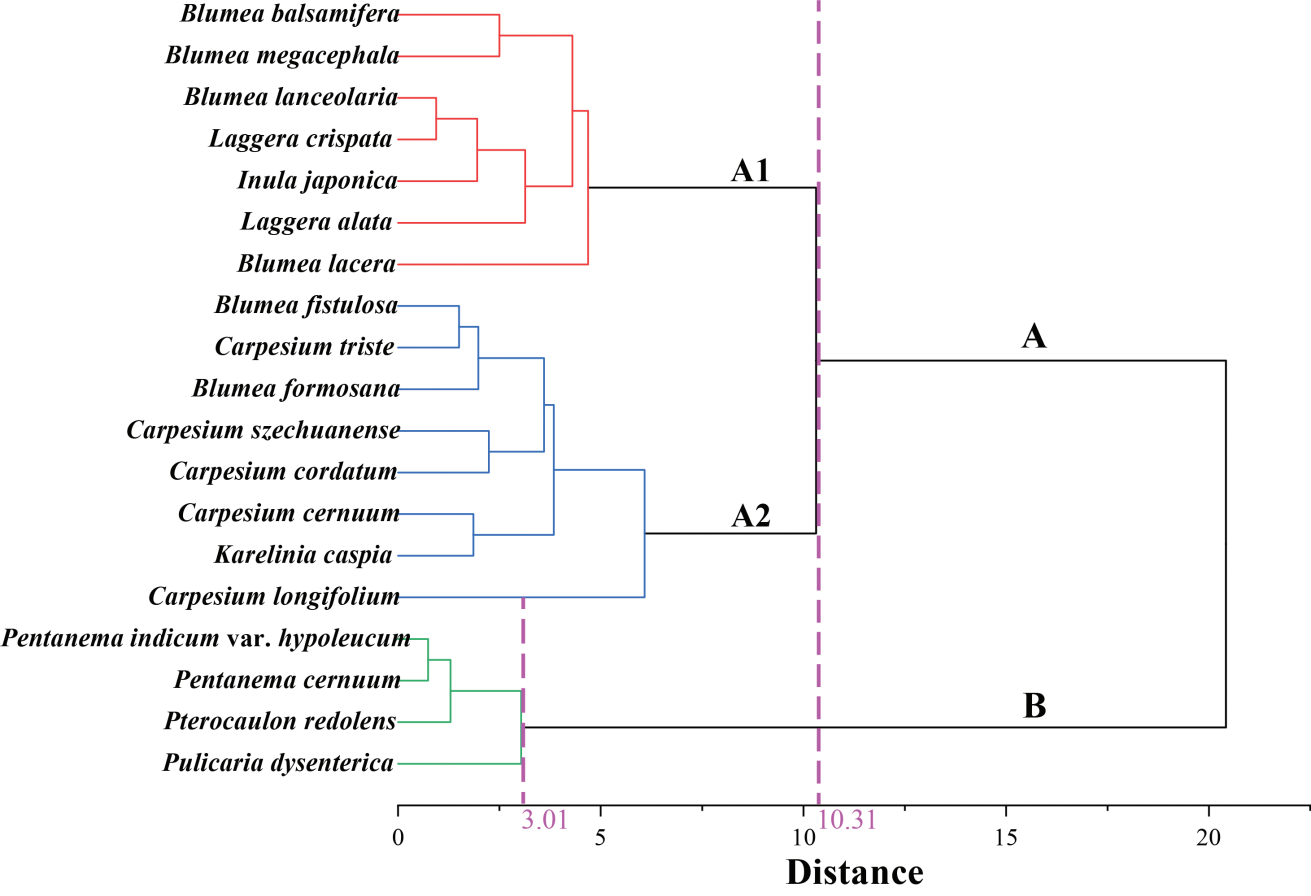
Cluster diagram (Ward’s method) of 19 Inuleae species based on ten quantitative pollen traits.

The palynological groups of the species were evaluated using hierarchical cluster analysis based on their morphological traits. The analysis separated the 19 Inuleae species into two major groups, Group A and Group B, with Group A further dividing into subgroups A1 and A2 (Fig. 9). The Euclidean distances between Group A and Group B were 10.31 and 3.01, respectively. Group A included 15 species, with subgroup A1 consisting of 7 species, such as *Blumea balsamifera*, *B. megacephala*, and *Inula japonica*, while subgroup A2 comprised 8 species, including all 5 *Carpesium* species, along with *B. fistulosa*, *B. formosana*, and *Karelinia caspia*. Group B consisted of 4 species: Pentanema indicum var. hypoleucum, *P. cernuum*, *Pterocaulon redolens*, and *Pulicaria dysenterica*.

## ﻿Discussion

This study documented substantial inter-individual variation in pollen morphology across 19 Inuleae species, particularly in grain size and exine ornamentation. All species exhibited spherical pollen grains with tricolporate apertures, and the exine surfaces were echinate. Variations in spine morphology provided measurable differences between species, consistent with previous studies on Asteraceae (Skvarla et al. 1977; Osman 2006; Pereira Coutinho and Dinis 2007; Coutinho et al. 2011; Peng et al. 2023). These traits contribute meaningfully to species delimitation and lay a morphological foundation for future taxonomic refinements within the tribe (Bahadur et al. 2022; Huang et al. 2023; Lin et al. 2023; Qu et al. 2025).

A comparative review was conducted to evaluate the consistency of morphological traits across genera. Among the *Blumea* species—*B. balsamifera*, *B. megacephala*, *B. lacera*, and *B. fistulosa*—we observed tricolporate pollen with echinate ornamentation, consistent with the descriptions in Peng et al. (2023). Our measurements of P and E were generally larger than those reported in their study, although the P/E ratios were similar. Since their study lacked illustrations, these results should be further validated. The pollen morphology of *Laggera crispata*, for which data are reported for the first time, showed subtle differences when compared to *L. alata*, as reported by Meo and Khan (2009). Minor variations in P and E were noted, likely reflecting genetic diversity, environmental adaptation, or methodological differences in pollen processing, as previously suggested (Mo et al. 1997; Zhang and Qian 2011; Lu et al. 2022). The pollen traits of *Inula japonica* conform to the morphological range established by Karlıoğlu Kılıç et al. (2021) for the genus *Inula*. Similarly, *Pulicaria dysenterica* shows patterns consistent with observations by Coutinho et al. (2011), supporting trait stability. The morphology of *Karelinia caspia*, the only species in its monotypic genus, corresponds to data reported by Lu et al. (2018). These consistencies support the reliability of our measurements. In the genus *Carpesium*, in addition to *C. cernuum*, whose pollen morphology was studied by Zarin et al. (2010), we provide the first descriptions for *C. szechuanense*, *C. triste*, *C. cordatum*, and *C. longifolium*, contributing to the understanding of pollen morphology within the genus. Additionally, this study establishes the first pollen morphological dataset for the genus *Pentanema* and extends previous work on *Pterocaulon* (Corrêa et al. 2008) by offering quantitative data on *P. redolens*, transitioning from qualitative to quantitative documentation.

The genera *Blumea* DC. and *Carpesium* L. are major members of the subtribe Inulinae within Inuleae. *Blumea* is the largest genus in the tribe (Anderberg 1991), predominantly consisting of herbs or shrubs, with the highest diversity in the tropics of the Old World (Chen and Anderberg 2011a; Pornpongrungrueng et al. 2016). Many species have notable economic and ecological value, with over half possessing medicinal or ethnobotanical uses (Jia and Li 2005; Zhang et al. 2019). However, taxonomic confusion persists due to limited morphological studies (Gagnepain 1920; Pornpongrungrueng et al. 2007, 2016). *Carpesium*, with approximately 20 species (Zhang et al. 2015), is distributed in Asia and Europe, particularly in mountainous regions of Southwest China, where several species are endemic (Chen and Anderberg 2011b). The genus is also valued for its medicinal applications in traditional herbal medicine (Kim et al. 1997; Moon 2007; Chung et al. 2008; Moon and Zee 2011), but its palynology remains understudied (Zarin et al. 2010; Peng et al. 2023). Comparative analysis reveals distinct differences in pollen morphology between the two genera. The maximum polar length (P) in *Blumea* reaches 32.90 µm and equatorial width (E) 29.79 µm, while in *Carpesium*, P is 35.06 µm and E is 32.42 µm. Pollen length (L) in *Blumea* ranges from 27.07 to 30.35 µm, whereas in *Carpesium* it ranges from 30.16 to 33.49 µm. These measurements indicate that *Blumea* pollen grains are generally smaller. Differences in exine ornamentation are useful for species identification, though further study of additional species is necessary for confirmation. The spine base diameter (D) in *Blumea* ranges from 3.00 to 3.69 µm, with spine height (H) from 3.58 to 4.09 µm, yielding a D/H ratio of 0.75 to 0.95. In *Carpesium*, while D (2.96 to 3.75 µm) is similar, H is higher (3.74 to 5.17 µm), resulting in a lower D/H ratio (0.68 to 0.80) and more slender spines. Furthermore, spine spacing (Ss) in *Blumea* is narrower (4.79 to 6.01 µm) compared to *Carpesium* (5.08 to 6.34 µm), reflecting a denser ornamentation pattern. These pollen traits provide morphological evidence that may aid in distinguishing the two genera, although further study is needed to confirm their diagnostic value in generic delimitation within Inuleae.

PCA highlighted pollen size and exine ornamentation as key contributors to morphological differentiation among species (Wang et al. 2009; Zhang et al. 2017; Maciejewska-Rutkowska et al. 2021; Jardine et al. 2022). PC1 (67.39% variance) mainly reflects size-related traits, while PC2 (13.67%) captures variation in exine ornamentation. Species on the positive PC1 axis, such as *C. longifolium* and *C. cernuum*, exhibit larger pollen grains. In contrast, species on the negative axis, such as *Pulicaria dysenterica* and *Pentanema cernuum*, show smaller pollen. PC2 further separates species by exine ornamentation, especially D/H and T/L ratios. For instance, *B. megacephala* and *B. balsamifera* have high D/H and T/L ratios, which may enhance pollen rigidity under arid or stressful conditions (Wodehouse 1935; Zhang and Zhou 2016). Conversely, *C. szechuanense* and *C. triste* occupy negative PC2 positions, with distinct ornamentation patterns possibly reflecting environmental adaptations (Tanaka et al. 2004; Ejsmond et al. 2011; Ďurišová et al. 2023).

According to Fu et al. (2016), the Inuleae tribe is divided into two subtribes, Inulinae and Plucheinae. The 19 species in this study belong to these two subtribes (Table 1). To explore the role of pollen traits in species differentiation, we performed hierarchical cluster analysis. The HCA results divided the 19 species into two main groups (Group A and Group B), with Group A further split into A1 and A2 subgroups. This division primarily aligns with a pollen size gradient, as strongly indicated by PC1 (Fig. 8), but also reveals intriguing patterns when compared to phylogenetic classifications. Group A1 includes species from *Blumea*, *Laggera*, and *Inula*. Nylinder and Anderberg (2015) noted that the boundaries between *Blumea* and *Laggera* are unclear, and some species show high morphological similarity. Our palynological data provide quantitative support for this observation, as these genera share intermediate values in key traits like Ss and D/H ratio (Table 2), which may explain their clustering in the HCA. Group A2 includes all *Carpesium* species, along with *B. fistulosa*, *B. formosana*, and *Karelinia caspia* (Plucheinae). The clustering of all five *Carpesium* species suggests strong palynological consistency within the genus. The placement of *K. caspia* here is incongruent with its subtribal classification but is robustly supported by shared traits like large pollen size and wide spine spacing (Table 2), highlighting the complex role of pollen evolution in taxonomy. Group B consists of four species. The two *Pentanema* species are clustered together, while the *Pterocaulon* and *Pulicaria* species are in separate branches. These species have smaller pollen sizes, with P values ranging from 22.64 to 23.85 µm. PCA analysis supports this, with Group B species primarily located in the negative region of the PC1 axis (Fig. 8). The tight clustering of the two *Pentanema* species, in particular, underscores the utility of pollen morphology for delimiting closely related species. These results suggest that pollen size is a key feature for species differentiation (Lu et al. 2009; Chung et al. 2010; Usma et al. 2022). Taken together, the HCA results are largely consistent with the groupings suggested by PCA, confirming the role of pollen traits in species differentiation. However, they do not fully align with molecular phylogenetic groups (Lin et al. 2023), and the incongruence in plant systematics may need integrative taxonomic approaches (Keating et al. 2023). PCA identified pollen size and exine ornamentation as the most influential traits, while HCA revealed species groupings based on overall pollen morphological similarity. Together, they confirm the significant value of pollen morphological data for the classification within Inuleae.

Quantitative pollen morphological data from 19 Inuleae species provide valuable insights into the systematic palynology of Asteraceae. In this study, one specimen per species was analyzed, a common practice in palynological research (Bahadur et al. 2022; Lu et al. 2022; Peng et al. 2023). While this approach facilitates interspecific comparisons, future studies could expand sampling to better assess the range and consistency of pollen traits, including ploidy counting and the evaluation of defective or sterile pollen. To capture potential nonlinear variation, dimensionality reduction techniques such as UMAP may complement PCA (McInnes et al. 2018; Becht et al. 2019; Erb et al. 2024). Higher-resolution imaging methods, such as transmission electron microscopy (TEM) combined with SEM, could resolve fine-scale exine structures (Jia et al. 2017; Gabarayeva et al. 2024). Combining morphological, genomic, and transcriptomic data may reveal the genetic basis of pollen variation and its evolutionary significance (Keating et al. 2023; Qu et al. 2025). These approaches could refine species delimitation and clarify pollen evolution within a broader phylogenetic context.

## ﻿Conclusion

This study documented variation in pollen morphology among 19 Inuleae species, focusing on grain size, shape, and exine ornamentation. These traits proved useful in distinguishing species and contributed to taxonomic classification. While HCA and PCA refined species clustering based on pollen traits, the taxonomic relationships among species cannot be fully clarified by pollen morphology alone. These results provide a foundation for future research on the role of pollen morphology in species differentiation within Inuleae.

## ﻿Additional information

### Use of AI

No use of AI was reported.

### Author ORCIDs

Tianmeng Qu https://orcid.org/0009-0008-0185-5306

Xinyu Chen https://orcid.org/0009-0008-6347-2490

Xinyi Zheng https://orcid.org/0009-0003-2300-5525

Yanru Zhang https://orcid.org/0009-0009-0450-2001

Yizhen Shao https://orcid.org/0000-0002-8865-3959

Hongze Sun https://orcid.org/0009-0000-5831-9979

Bing Zhang https://orcid.org/0009-0006-6019-0629

Gan Xie https://orcid.org/0000-0002-8265-1565

Zhixi Fu https://orcid.org/0000-0002-2789-6287

### Data availability

All of the data that support the findings of this study are available in the main text or Supplementary Information.

## Supplementary materials

Supplementary material 1 Pollen morphological descriptions of 19 species from 8 genera of Inuleae Data type docx https://binary.pensoft.net/file/1433377This dataset is made available under the Open Database License (http://opendatacommons.org/licenses/odbl/1.0/). The Open Database License (ODbL) is a license agreement intended to allow users to freely share, modify, and use this Dataset while maintaining this same freedom for others, provided that the original source and author(s) are credited.Tianmeng Qu
