# Supplementary material for: ﻿Pollen morphology and species differentiation in selected species of Inuleae (Asteraceae)
Source: PhytoKeys. 2025 Oct 8;263:215–40. doi: 10.3897/phytokeys.263.165364 (PMC12529077; doi:10.3897/phytokeys.263.165364)
Supplement: Supplementary material 1 — Pollen morphological descriptions of 19 species from 8 genera of Inuleae [file phytokeys-263-215_article-165364__-s001.docx]

Supplementary File

Pollen morphological descriptions of 19 species from 8 genera of Inuleae. Pollen morphology of Inuleae: pollen grains spherical; almost circular in equatorial view and trilobate circular in polar view; tricolporate apertures with a granulate aperture membrane; the exine ornamentation is spinose (LM), echinate (SEM), most spine shapes are tapering to a sharp point, with one or more rows of perforations at the base, and the inter-spinal area is perforate.

**1 Genus: *Blumea* DC.**

**1.1 Investigated species: *Blumea balsamifera* (L.) DC. (Table 2, Fig. 2A, Fig. 3A, Fig. 4A1–A4)**

Pollen grains spherical. Almost circular in equatorial view and trilobate circular in polar view. Tricolporate apertures with a granulate aperture membrane. Polar length (P) = 28.82 ± 1.48 µm (M ± SD), Equatorial width (E) = 26.86 ± 1.12 µm (M ± SD), P/E = 1.07 ± 0.04 (M ± SD), Exine thickness (T) = 4.27 ± 0.27 µm (M ± SD), and Pollen length (L) = 28.04 ± 1.29 µm (M ± SD), T/L = 0.15 ± 0.01 (M ± SD). The exine ornamentation is spinose (LM), echinate (SEM). Under SEM, Diameter of spine base (D) = 3.39 ± 0.12 µm (M ± SD), Spine height (H) = 3.58 ± 0.17 µm (M ± SD), D/H = 0.95 ± 0.03 (M ± SD), and Spine spacing (Ss) = 4.79 ± 0.23 µm (M ± SD). Spine tapering to a sharp point, with 2-3 rows of perforations at the base, and the inter-spinal area is perforate.

**1.2 Investigated species: *Blumea megacephala* (Randeria) C.T.Chang & C.H.Yu (Table 2, Fig. 2B, Fig. 3B, Fig. 4B1–B4)**

Pollen grains spherical. Almost circular in equatorial view and trilobate circular in polar view. Tricolporate apertures with a granulate aperture membrane. P = 26.68 ± 1.66 µm, E = 24.76 ± 1.21 µm, P/E = 1.08 ± 0.04, T = 4.10 ± 0.30 µm, L = 27.50 ± 1.29 µm, T/L = 0.15 ± 0.01. The exine ornamentation is spinose (LM), echinate (SEM). Under SEM, D = 3.69 ± 0.36 µm, H = 3.95 ± 0.36 µm, D/H = 0.93 ± 0.04, Ss = 6.01 ± 0.55 µm. Spine tapering to a blunt tip, with 3-4 rows of perforations at the base, and the inter-spinal area is perforate.

**1.3 Investigated species: *Blumea lacera* (Burm.f.) DC. (Table 2, Fig. 2C, Fig. 3C, Fig. 4C1–C4)**

Pollen grains spherical. Almost circular in equatorial view and trilobate circular in polar view. Tricolporate apertures with a granulate aperture membrane. P = 25.70 ± 1.48 µm, E = 25.54 ± 0.97 µm, P/E = 1.01 ± 0.05, T = 4.09 ± 0.26 µm, L = 27.07 ± 1.57 µm, T/L = 0.15 ± 0.01. The exine ornamentation is spinose (LM), echinate (SEM). Under SEM, D = 3.00 ± 0.17 µm, H = 3.98 ± 0.23 µm, D/H = 0.75 ± 0.04, Ss = 4.80 ± 0.43 µm. Spine tapering to a sharp point, with 3 rows of perforations at the base, and the inter-spinal area is perforate.

**1.4 Investigated species: *Blumea fistulosa* (Roxb.) Kurz (Table 2, Fig. 2D, Fig. 3D, Fig. 4D1–D4)**

Pollen grains spherical. Almost circular in equatorial view and trilobate circular in polar view. Tricolporate apertures with a granulate aperture membrane. P = 29.94 ± 1.67 µm, E = 27.58 ± 1.58 µm, P/E = 1.09 ± 0.03, T = 4.05 ± 0.18 µm, L = 30.20 ± 1.10 µm, T/L = 0.13 ± 0.00. The exine ornamentation is spinose (LM), echinate (SEM). Under SEM, D = 3.31 ± 0.34 µm, H = 3.86 ± 0.40 µm, D/H = 0.86 ± 0.06, Ss = 4.92 ± 0.42 µm. Spine tapering to a sharp point, with 2 rows of perforations at the base, and the inter-spinal area is perforate.

**1.5 Investigated species: *Blumea lanceolaria* (Roxb.) Druce (Table 2, Fig. 2E, Fig. 3E, Fig. 4E1–E4)**

Pollen grains spherical. Almost circular in equatorial view and trilobate circular in polar view. Tricolporate apertures with a granulate aperture membrane. P = 27.72 ± 0.67 µm, E = 25.73 ± 0.64 µm, P/E = 1.08 ± 0.02, T = 3.96 ± 0.23 µm, L = 28.23 ± 1.60 µm, T/L = 0.14 ± 0.01. The exine ornamentation is spinose (LM), echinate (SEM). Under SEM, D = 3.13 ± 0.27 µm, H = 3.61 ± 0.24 µm, D/H = 0.87 ± 0.04, Ss = 5.41 ± 0.39 µm. Spine tapering to a blunt tip, with 2 rows of perforations at the base, and the inter-spinal area is perforate.

**1.6 Investigated species: *Blumea formosana* Kitam. (Table 2, Fig. 2F, Fig. 3F, Fig. 4F1–F4)**

Pollen grains spherical. Almost circular in equatorial view and trilobate circular in polar view. Tricolporate apertures with a granulate aperture membrane. P = 32.90 ± 1.66 µm, E = 29.79 ± 1.50 µm, P/E = 1.10 ± 0.02, T = 4.14 ± 0.13 µm, L = 30.35 ± 1.37 µm, T/L = 0.14 ± 0.01. The exine ornamentation is spinose (LM), echinate (SEM). Under SEM, D = 3.26 ± 0.19 µm, H = 4.09 ± 0.24 µm, D/H = 0.80 ± 0.03, Ss = 5.69 ± 0.24 µm. Spine tapering to a blunt tip, with 2-3 rows of perforations at the base, and the inter-spinal area is perforate.

**2 Genus: *Carpesium* L.**

**2.1 Investigated species: *Carpesium szechuanense* F.H.Chen & C.M.Hu (Table 2, Fig. 2G, Fig. 3G, Fig. 5A1–A4)**

Pollen grains spherical. Almost circular in equatorial view and trilobate circular in polar view. Tricolporate apertures with a granulate aperture membrane. P = 31.43 ± 0.97 µm, E = 28.86 ± 0.90 µm, P/E = 1.09 ± 0.02, T = 4.53 ± 0.19 µm, L = 31.55 ± 1.41 µm, T/L = 0.14 ± 0.00. The exine ornamentation is spinose (LM), echinate (SEM). Under SEM, D = 2.96 ± 0.22 µm, H = 4.34 ± 0.33 µm, D/H = 0.68 ± 0.03, Ss = 5.23 ± 0.44 µm. Spine tapering to a sharp point, with 1-2 rows of perforations at the base, and the inter-spinal area is perforate.

**2.2 Investigated species: *Carpesium triste* Maxim. (Table 2, Fig. 2H, Fig. 3H, Fig. 5B1–B4)**

Pollen grains spherical. Almost circular in equatorial view and trilobate circular in polar view. Tricolporate apertures with a granulate aperture membrane. P = 31.60 ± 0.83 µm, E = 29.12 ± 0.70 µm, P/E = 1.09 ± 0.02, T = 4.06 ± 0.21 µm, L = 30.16 ± 1.55 µm, T/L = 0.13 ± 0.00. The exine ornamentation is spinose (LM), echinate (SEM). Under SEM, D = 2.96 ± 0.31 µm, H = 3.74 ± 0.38 µm, D/H = 0.79 ± 0.04, Ss = 5.08 ± 0.27 µm. Spine tapering to a sharp point, with 1-2 rows of perforations at the base, and the inter-spinal area is perforate.

**2.3 Investigated species: *Carpesium cordatum* F.H.Chen & C.M.Hu (Table 2, Fig. 2I, Fig. 3I, Fig. 5C1–C4)**

Pollen grains spherical. Almost circular in equatorial view and trilobate circular in polar view. Tricolporate apertures with a granulate aperture membrane. P = 34.34 ± 1.00 µm, E = 32.16 ± 0.97 µm, P/E = 1.07 ± 0.01, T = 4.66 ± 0.12 µm, L = 33.49 ± 0.84 µm, T/L = 0.14 ± 0.00. The exine ornamentation is spinose (LM), echinate (SEM). Under SEM, D = 3.16 ± 0.16 µm, H = 4.11 ± 0.22 µm, D/H = 0.77 ± 0.04, Ss = 5.29 ± 0.23 µm. Spine tapering to a sharp point, with 1-2 rows of perforations at the base, and the inter-spinal area is perforate.

**2.4 Investigated species: *Carpesium cernuum* L. (Table 2, Fig. 2J, Fig. 3J, Fig. 5D1–D4)**

Pollen grains spherical. Almost circular in equatorial view and trilobate circular in polar view. Tricolporate apertures with a granulate aperture membrane. P = 31.73 ± 0.82 µm, E = 28.82 ± 0.66 µm, P/E = 1.10 ± 0.02, T = 4.56 ± 0.17 µm, L = 30.95 ± 1.13 µm, T/L = 0.15 ± 0.00. The exine ornamentation is spinose (LM), echinate (SEM). Under SEM, D = 3.47 ± 0.13 µm, H = 4.32 ± 0.24 µm, D/H = 0.80 ± 0.04, Ss = 5.70 ± 0.32 µm. Spine tapering to a sharp point, with 2 rows of perforations at the base, and the inter-spinal area is perforate.

**2.5 Investigated species: *Carpesium longifolium* F.H.Chen & C.M.Hu (Table 2, Fig. 2K, Fig. 3K, Fig. 5E1–E4)**

Pollen grains spherical. Almost circular in equatorial view and trilobate circular in polar view. Tricolporate apertures with a granulate aperture membrane. P = 35.06 ± 1.41 µm, E = 32.42 ± 1.70 µm, P/E = 1.08 ± 0.03, T = 5.12 ± 0.18 µm, L = 32.98 ± 1.20 µm, T/L = 0.16 ± 0.01. The exine ornamentation is spinose (LM), echinate (SEM). Under SEM, D = 3.75 ± 0.35 µm, H = 5.17 ± 0.55 µm, D/H = 0.73 ± 0.04, Ss = 6.34 ± 0.51 µm. Spine tapering to a sharp point, with 2 rows of perforations at the base, and the inter-spinal area is perforate.

**3 Genus: *Inula* L.**

**3.1 Investigated species: *Inula japonica* Thunb. (Table 2, Fig. 2L, Fig. 3L, Fig. 5F1–F4)**

Pollen grains spherical. Almost circular in equatorial view and trilobate circular in polar view. Tricolporate apertures with a granulate aperture membrane. P = 27.51 ± 0.74 µm, E = 26.23 ± 0.91 µm, P/E = 1.05 ± 0.02, T = 3.86 ± 0.13 µm, L = 27.06 ± 1.04 µm, T/L = 0.14 ± 0.00. The exine ornamentation is spinose (LM), echinate (SEM). Under SEM, D = 2.90 ± 0.24 µm, H = 3.51 ± 0.27 µm, D/H = 0.83 ± 0.03, Ss = 5.06 ± 0.33 µm. Spine tapering to a sharp point, with one row of perforations at the base, and the inter-spinal area is perforate.

**4 Genus: *Karelinia* Less.**

**4.1 Investigated species: *Karelinia caspia* (Pall.) Less. (Table 2, Fig. 2M, Fig. 3M, Fig. 6A1–A4)**

Pollen grains spherical. Almost circular in equatorial view and trilobate circular in polar view. Tricolporate apertures with a granulate aperture membrane. P = 31.28 ± 0.81 µm, E = 27.69 ± 0.90 µm, P/E = 1.13 ± 0.02, T = 4.33 ± 0.21 µm, L = 29.65 ± 1.19 µm, T/L = 0.15 ± 0.00. The exine ornamentation is spinose (LM), echinate (SEM). Under SEM, D = 3.28 ± 0.13 µm, H = 3.79 ± 0.17 µm, D/H = 0.87 ± 0.03, Ss = 5.62 ± 0.23 µm. Spine tapering to a sharp point, with 2 rows of perforations at the base, and the inter-spinal area is perforate.

**5 Genus: *Laggera* Sch.Bip. ex Benth. & Hook.f.**

**5.1 Investigated species: *Laggera crispata* (Vahl) Hepper & J.R.I.Wood (Table 2, Fig. 2N, Fig. 3N, Fig. 6B1–B4)**

Pollen grains spherical. Almost circular in equatorial view and trilobate circular in polar view. Tricolporate apertures with a granulate aperture membrane. P = 27.19 ± 1.20 µm, E = 25.51 ± 1.15 µm, P/E = 1.07 ± 0.02, T = 3.94 ± 0.21 µm, L = 27.97 ± 0.82 µm, T/L = 0.14 ± 0.01. The exine ornamentation is spinose (LM), echinate (SEM). Under SEM, D = 3.27 ± 0.19 µm, H = 3.83 ± 0.14 µm, D/H = 0.85 ± 0.03, Ss = 5.76 ± 0.17 µm. Spine tapering to a sharp point, with 1-2 rows of perforations at the base, and the inter-spinal area is perforate.

**5.2 Investigated species: *Laggera alata* (D. Don) Sch.Bip. ex Oliv. (Table 2, Fig. 2O, Fig. 3O, Fig. 6C1–C4)**

Pollen grains spherical. Almost circular in equatorial view and trilobate circular in polar view. Tricolporate apertures with a granulate aperture membrane. P = 27.19 ± 1.42 µm, E = 24.51 ± 1.33 µm, P/E = 1.11 ± 0.03, T = 3.75 ± 0.22 µm, L = 26.62 ± 0.93 µm, T/L = 0.14 ± 0.01. The exine ornamentation is spinose (LM), echinate (SEM). Under SEM, D = 2.75 ± 0.16 µm, H = 3.22 ± 0.13 µm, D/H = 0.85 ± 0.03, Ss = 4.65 ± 0.26 µm. Spine tapering to a sharp point, with 2 rows of perforations at the base, and the inter-spinal area is perforate.

**6 Genus: *Pentanema* Cass.**

**6.1 Investigated species: *Pentanema indicum* var. *hypoleucum* (Hand.-Mazz.) Y.Ling (Table 2, Fig. 2P, Fig. 3P, Fig. 6D1–D4)**

Pollen grains spherical. Almost circular in equatorial view and trilobate circular in polar view. Tricolporate apertures with a granulate aperture membrane. P = 23.51 ± 0.91 µm, E = 22.39 ± 0.75 µm, P/E = 1.05 ± 0.03, T = 3.18 ± 0.19 µm, L = 23.26 ± 0.73 µm, T/L = 0.14 ± 0.01. The exine ornamentation is spinose (LM), echinate (SEM). Under SEM, D = 2.52 ± 0.18 µm, H = 2.91 ± 0.24 µm, D/H = 0.87 ± 0.02, Ss = 4.38 ± 0.27 µm. Spine tapering to a blunt tip, with 1-2 rows of perforations at the base, and the inter-spinal area is perforate.

**6.2 Investigated species: *Pentanema cernuum* (Dalzell) Y.Ling (Table 2, Fig. 2Q, Fig. 3Q, Fig. 6E1–E4)**

Pollen grains spherical. Almost circular in equatorial view and trilobate circular in polar view. Tricolporate apertures with a granulate aperture membrane. P = 22.64 ± 0.92 µm, E = 21.36 ± 0.82 µm, P/E = 1.06 ± 0.03, T = 3.20 ± 0.14 µm, L = 23.05 ± 1.06 µm, T/L = 0.14 ± 0.01. The exine ornamentation is spinose (LM), echinate (SEM). Under SEM, D = 2.60 ± 0.10 µm, H = 2.96 ± 0.14 µm, D/H = 0.88 ± 0.03, Ss = 4.51 ± 0.18 µm. SSpine tapering to a sharp point, with 2 rows of perforations at the base, and the inter-spinal area is perforate.

**7 Genus: *Pterocaulon* Elliott**

**7.1 Investigated species: *Pterocaulon redolens* (Willd.) Fern.**-**Vill. (Table 2, Fig. 2R, Fig. 3R, Fig. 6F1–F4)**

Pollen grains spherical. Almost circular in equatorial view and trilobate circular in polar view. Tricolporate apertures with a granulate aperture membrane. P = 22.77 ± 1.56 µm, E = 22.00 ± 1.52 µm, P/E = 1.04 ± 0.02, T = 3.06 ± 0.12 µm, L = 22.67 ± 1.31 µm, T/L = 0.14 ± 0.01. The exine ornamentation is spinose (LM), echinate (SEM). Under SEM, D = 2.42 ± 0.10 µm, H = 2.69 ± 0.11 µm, D/H = 0.90 ± 0.02, Ss = 4.42 ± 0.21 µm. Spine tapering to a blunt tip, with 1-2 rows of perforations at the base, and the inter-spinal area is perforate.

**8 Genus: *Pulicaria* Gaertn.**

**8.1 Investigated species: *Pulicaria dysenterica* (L.) Bernh. (Table 2, Fig. 2S, Fig. 3S, Fig. 6G1–G4)**

Pollen grains spherical. Almost circular in equatorial view and trilobate circular in polar view. Tricolporate apertures with a granulate aperture membrane. P = 23.85 ± 1.22 µm, E = 22.29 ± 1.18 µm, P/E = 1.07 ± 0.02, T = 3.06 ± 0.18 µm, L = 23.69 ± 1.68 µm, T/L = 0.13 ± 0.01. The exine ornamentation is spinose (LM), echinate (SEM). Under SEM, D = 2.56 ± 0.16 µm, H = 3.32 ± 0.13 µm, D/H = 0.77 ± 0.05, Ss = 4.22 ± 0.29 µm. Spine tapering to a sharp point, with one row of perforations at the base, and the inter-spinal area is perforate.
